# Current State of Orthobiologics in Treatment of Knee Osteoarthritis—Future Directions

**DOI:** 10.3390/ijms27114738

**Published:** 2026-05-25

**Authors:** Woojin Lee, Qing Zhao Ruan, Jamal J. Hasoon, Ronald J. Kulich, Timothy R. Deer, Dawood Sayed, Franzes Anne Z. Liongson, Elizabeth Hatfield, Maged Guirguis, Alan D. Kaye, Zachary L. McCormick, Robert Jason Yong, Christopher L. Robinson

**Affiliations:** 1 Warren Alpert Medical School, Brown University, Providence, RI 02912, USA; 2 Department of Anesthesia, Critical Care, and Pain Medicine, McGovern Medical School, UTHealth, Houston, TX 77030, USA; 3 Department of Anesthesia, Critical Care, and Pain Medicine, Massachusetts General Hospital, Boston, MA 02114, USA; 4 Department of Psychiatry, Massachusetts General Hospital, Boston, MA 02114, USA; 5 Spine and Nerve Center of the Virginias, West Virginia University, Health Sciences Campus, Charleston, WV 25301, USA; 6 Department of Anesthesiology, The University of Kansas School of Medicine, Kansas City, KS 66045, USA; 7 Department of Anesthesiology, Perioperative Care, and Pain Medicine, NYU Grossman School of Medicine, New York, NY 10016, USA; 8 Department of Oral & Maxillofacial Surgery/Hospital Dentistry, University of Michigan School of Dentistry, Ann Arbor, MI 48104, USA; 9 Department of Interventional Pain, Oschner Health System, New Orleans, LA 70115, USA; 10 Department of Anesthesiology, Louisiana State University Health Sciences Center Shreveport, Shreveport, LA 71103, USA; 11 University Orthopaedic Center, Salt Lake City, UT 84108, USA; 12 Department of Anesthesiology, Perioperative, and Pain Medicine, Brigham and Women’s Hospital, Harvard Medical School, Boston, MA 02115, USA; 13 Division of Pain Medicine, Department of Anesthesiology and Critical Care, The Johns Hopkins University School of Medicine, Baltimore, MD 21205, USA

**Keywords:** knee osteoarthritis, orthobiologics, regenerative medicine, platelet-rich plasma

## Abstract

As the population ages, the incidence and prevalence of musculoskeletal degeneration, such as osteoarthritis, increase. While the currently accepted treatment options provide symptomatic and functional improvement, they do not halt the progression of osteoarthritis. This results in the eventual need for surgery for many patients with advanced osteoarthritis. Due to the seemingly inevitable progression of OA, many clinicians and researchers have shifted their focus to regenerative therapies. Orthobiologics, a specific type of regenerative therapy designed to treat orthopedic conditions, has been gaining traction in recent years due to the utilization of autologous biological substances and synthetic peptides in healing musculoskeletal injuries and degenerative conditions. Orthobiologics can be distinguished into one of four classes: cell-based, biologic fluids-based, matrix-based, molecular-based, and based on their composition. In this review, key examples of each class, mechanism of action, and current clinical data for each agent are examined. Limitations of current orthobiologics involve a lack of standardization in the preparation and administration of each agent, as well as uniformity in assessment endpoints across different clinical studies. Lastly, we will discuss future directions of orthobiologics as a therapy for the treatment of osteoarthritis.

## 1. Introduction

Orthobiologics have been gaining popularity in recent years as both incidence and prevalence of joint osteoarthritis (OA) continue to increase globally [1,2,3,4]. From 1990 to 2020, the prevalence of OA rose by 132.2% while the age-standardized incidence rate increased from 233.0 to 255.0 per 100,000 worldwide [1]. This rise is expected to continue due to the increasing age of the population and longer life expectancy [1,2,3,4]. Orthobiologics, defined as biological substances utilized to promote healing in musculoskeletal injuries and degenerative conditions, provide symptomatic and functional improvements in degenerative conditions, such as OA [5,6]. Some orthobiologic agents have demonstrated potential regenerative effects by decelerating the progression of OA, as evidenced by imaging assessments [7,8,9,10,11,12,13]. An increasing number of preclinical and clinical trials are currently underway to substantiate these claims. Knee OA has been of particular interest in this ongoing research as it accounts for 60–85% of the total OA cases globally [3,4,14]. Current standard treatments for knee OA, including exercise, weight loss, NSAIDs, topical agents, corticosteroid or hyaluronic acid injections, and radiofrequency ablation, have not been effective in decreasing the incidence of knee OA, ultimately resulting in an increased need for surgical intervention such as total knee arthroplasty [15,16,17,18,19,20,21,22,23,24,25,26,27,28,29,30]. Therefore, if orthobiologics can sufficiently prove their regenerative effect on degenerative conditions such as knee OA, it may result in a regression of knee OA prevalence, resulting in a decreasing disease burden worldwide, providing better quality of life in the aging population, and significantly lowering medical costs in managing this condition.

With the growing interest in regenerative medicine, a broad spectrum of orthobiologic agents is currently under clinical investigation, while novel therapies continue to be developed in pursuit of meaningful regenerative potential. Given the expanding number and complexity of these agents, a composition-based classification system may provide a more structured framework for understanding and organizing the field. In this paper, we propose a classification system for orthobiologics, highlight representative agents within each category based on the depth of available clinical evidence, review the current clinical data surrounding these therapies, and discuss future directions in the evolving landscape of orthobiologic treatment for knee osteoarthritis.

## 2. Methods

The literature was identified through a comprehensive search of electronic databases, including PubMed, MEDLINE, and Google Scholar. A combination of free-text keywords and Medical Subject Headings (MeSH) was utilized to capture relevant studies. Search terms included ‘orthobiologics,’ ‘knee osteoarthritis,’ ‘treatment of knee osteoarthritis’, ‘pathophysiology of knee osteoarthritis, ‘incidence and prevalence of knee osteoarthritis’, ‘limitations of current treatment therapies for knee osteoarthritis’, ‘regenerative medicine,’ ‘cartilage regeneration’, ‘genetic expression of osteoarthritis’, ‘stem cells in degenerative joints’, ‘mechanisms of actions of orthobiologics’, ‘limitations of orthobiologics’, ‘MSC,’ ‘ACI,’ ‘PRP,’ ‘BMAC,’ ‘SVF,’ ‘MACI,’ ‘HA hydrogel,’ ‘AMM/ASA,’ ‘MFAT,’ ‘rFGF-18,’ ‘TPX-100,’ ‘lorecivivint,’ ‘LNA043,’ and ‘BPC-157.’

The article selection was conducted through initial abstract screening followed by a full-text review. The inclusion criteria focused on reviews, clinical studies, and trials evaluating orthobiologics and their efficacy in the treatment of knee osteoarthritis and cartilage defects. The key data extracted included patient-reported outcomes, histological and imaging evidence of cartilage regeneration, and reported adverse effects. The quality of the articles was assessed primarily by their study design and sample size, with priority given to single and double-blind randomized controlled studies and larger sample sizes. During the article selection, we noted a relative lack of high-quality studies for certain orthobiologic agents. Nevertheless, these studies, including smaller sample size cohort studies, were included because they remain important representatives of their respective orthobiologic classes with promising potential. Inclusion of these lower-quality studies was also intended to provide a more comprehensive and accurate representation of the current state of orthobiologic research. WL and CR were the primary authors involved in the study selection process, and WL was responsible for the final inclusion decision.

Eligible studies were limited to those published in English-language journals between 2005 and 2025, with select earlier landmark studies included to establish foundational concepts. Greater emphasis was placed on recent publications to ensure the incorporation of the most current clinical evidence and to provide a comprehensive overview of orthobiologic therapies. 

## 3. Classifications of Orthobiologics

Orthobiologics can be distinguished into one of four classes based on their composition: cell-based, biologic fluids-based, matrix-based, and molecular-based (Table 1 and Table 2). Cell-based orthobiologics are primarily composed of cells with regenerative properties, such as mesenchymal stromal cells (MSCs) or autologous chondrocyte implantation (ACI), which can be injected into ongoing OA to promote regrowth of articular cartilage. Biologic fluids-based orthobiologics are broader in classification in that they are concentrates of regenerative cells and proteins, such as growth factors (GF). Main examples in this class would be platelet-rich plasma (PRP), bone marrow aspirate concentrate (BMAC), and stromal vascular fraction (SVF) [5,6,31]. Matrix-based orthobiologics utilize scaffolds or matrices to deliver growth factors and cells, providing a structural framework for new tissue to grow on [32,33,34,35,36]. Molecular-based orthobiologics are primarily peptides, recombinant proteins, and growth factors that are delivered directly into the target site to amplify the body’s self-repair and regeneration. Some molecular-based orthobiologics have also been shown to exhibit regenerative and positive disease-modifying effects in OA. Key examples include recombinant fibroblast growth factor-18 (rFGF-18), TPX-100, lorecivivint (LOR), LNA043, a derivative of angiopoietin-like 3 (ANGPTL-3), and body protection compound-157 (BPC-157) [11,37,38,39,40,41,42].

## 4. Mechanism of Action and Efficacy Data on Orthobiologics

### 4.1. Cell-Based Orthobiologics

#### Mesenchymal Stromal Cells

MSCs are adult stem cells with promising potential in the treatment of knee OA primarily due to their differentiation capabilities, anti-inflammatory properties, and immunomodulatory effects. They are commonly collected from bone marrow and then isolated via density gradient centrifugation. Alternatively, they can also be collected and isolated via enzymatic digestion of the adipose tissue. They are further isolated and cultured by plating in culture vessels with a growth medium, maintained at 37 °C with 5% CO_2_. Then, they can be detached from the vessels, tagged with specific markers, such as CD73, CD90, or CD105, for further characterization via flow cytometry and preserved in liquid nitrogen [90].

It is thought that MSCs can differentiate into various cell types in response to local biochemical and biomechanical stress. They can be differentiated into chondrocytes via transforming growth factor beta (TGF-β) pathway to substantiate chondrocytes’ regenerative effect on hyaline cartilage turnover. They can also be differentiated into osteoblasts via the bone morphogenetic protein-2 (BMP-2) signaling pathway to assist in osseous turnover. If MSCs are introduced to a high-biomechanical-stress environment, such as in bone matrices, they are prone to osteogenic differentiation, whereas if they are exposed to a low-stress environment, they are more likely to undergo chondrogenic differentiation [5]. Once differentiated, these MSCs, which turned into chondrocytes, in the presence of chondroitin sulphate, can provide anti-inflammation and immunomodulation via suppressing pro-inflammatory pathways such as NF-κB and decreasing the release of inflammatory cytokines (IL-1β and TNF-α), while upregulating anti-inflammatory proteins, including TNF-α-induced protein 6 (TSG6) and thrombospondin-1 [55,91]. The thought is that this will keep the vicious cycle of inflammation in the knee OA under control, nurturing a healing environment.

MSCs from bone marrow and synovium are especially promising because they specifically exhibited increased differentiation into chondrocytes compared to MSCs from other sources in vitro. In addition, if a parathyroid hormone-like peptide (PTHrP) and a basic fibroblast growth factor (bFGF) are added with MSCs, there is suppression in excessive chondrogenic differentiation and synthesis of collagen X, keeping the process regulated [43].

These in vitro and ex vivo studies have shown that MSCs can remain viable for a prolonged period and retain their differentiating capabilities under various circumstances [5]. Multiple randomized controlled trials (RCTs) have been performed since then, comparing MSCs to other treatment modalities such as HA, CS, and a placebo. A recent meta-analysis by Tabet et al. across 25 RCTs with 1048 patients revealed an improvement in the pain visual analog scale (VAS) in patients with knee OA with advanced MSC therapy compared to those who underwent viscosupplementation for up to 12 months. However, due to inconsistent inclusion criteria and primary endpoint assessments, the evidence was considered uncertain [44]. A 2025 meta-analysis by Cao et al. across 502 patients with knee OA using eight RCTs revealed more reliable evidence, with an improvement seen in 6 and 12 months on the Western Ontario and McMaster Universities Osteoarthritis Index (WOMAC), VAS, and Knee Injury and Osteoarthritis Outcome Score (KOOS) with MSC treatment compared to the control group, which found no significant differences in adverse effects [45]. Due to dependency on local growth factors and cytokines, MSCs as the sole treatment of knee OA have not been as clinically popular compared to biologic fluids-based orthobiologics, such as PRP and BMAC, which already contain a variety of autologous growth factors and bioactive molecules crucial in the regenerative process.

## 5. Autologous Chondrocyte Implantation

ACI is a procedure that entails harvesting autologous chondrocytes, often through joint arthroscopic biopsy, which are then cultured and multiplied to be reimplanted into the damaged articular cartilage. The reimplantation occurs through arthrotomy, where another harvested patch of tissue, such as periosteum from proximal tibia or distal femur, is sewn over the defect, and the cultured chondrocytes are injected under the membrane. The idea is that these additional chondrocytes will aid in repairing articular cartilage defects before OA progresses [46].

In the past decade, ACI has been utilized as a treatment option primarily for young patients with isolated articular cartilage defects with minimal osteoarthritic changes to decrease the possibility of a premature total knee replacement. Ideal candidates are young patients with focal defects that can be covered with a harvested patch of tissue for maximum efficacy. ACI is not commonly used in patients with widespread or advanced OA, as the membrane or chondrocytes underneath would not be able to provide a joint-wide regenerative effect [46]. ACI is also not a recommended procedure for elderly patients due to decreased reparative ability of autologous chondrocytes [72].

In young patients with limited cartilage defects and early OA changes, ACI provided significant improvement in pain and function per WOMAC subscales with a decreased need for joint replacement at the 5-year follow-up [46]. A more recent systematic review by Colombini et al. revealed that ACI, which was commonly used in patients with K-L grade 1 OA, provided sustained clinical improvement up to 11 years with a failure rate of approximately 10% [47]. Despite evidence of clinical efficacy with increasing utilization of ACI, reoperation rates remain high due to graft hypertrophy [48]. With additional chondrocytes being implanted, there is a lack of regulation in collagen production, resulting in arthrofibrosis and periosteal hypertrophy [46]. This complication warrants further operations, including chondroplasty, meniscectomy, and microfracture [48]. Currently, the available clinical evidence level for ACI is IV and requires more structured, comparative studies, such as RCTs, to establish its clinical relevance.

## 6. Biologic Fluids-Based Orthobiologics

### Platelet-Rich Plasma

PRP is an autologous concentrate of platelets and is plasma-rich in growth factors and cytokines that stimulate the repair of damaged tissues and joints. It is utilized in the treatment of multiple conditions, including OA, tendinopathy, myofascial injuries, and even androgenic alopecia. PRP is commonly prepared autologously by drawing blood from the patient. Then, 30–60 milliliters (mL) is collected in a sterile manner, followed by two rounds of centrifugation to separate the plasma, platelets, and red blood cells. The blood is mixed with an anticoagulant to prevent premature clotting. Platelet-poor plasma, which is concentrated at the top, is removed, and the remaining PRP is then collected to be used immediately for maximum efficacy [49]. Additional steps, such as leukoreduction filtration or density gradient centrifugation, are utilized to create leukocyte-poor PRP, which has been shown to be more effective in treating OA as it causes less inflammation and pain compared to leukocyte-rich PRP [92].

In case of knee OA, PRP is injected intra-articularly, where activated platelets release α-granules containing growth factors, including TGF-β, vascular endothelial growth factor (VEGF), platelet-derived growth factor (PDGF), insulin-like growth factor (IGF), and FGF, to accelerate the healing process. More specifically, these growth factors stimulate local cells, including chondrocytes, to produce new collagen, promote angiogenesis for increased nutrient supply and cellular migration, induce anti-inflammatory effects, and provide chondroprotection. This allows PRP to stand out amongst other commonly used anti-inflammatory treatments, including systemic NSAIDs and intra-articular CS.

A meta-analysis containing 15 RCTs and a sample size of 1314 participants by Han et al. compared PRP to HA injections, which has shown superiority in WOMAC pain, stiffness, and function subscales, with a VAS pain score taken at 12 months post-injection with no statistical difference in adverse events [50]. More recent meta-analysis of 3104 participants using 35 RCTs by Qiao et al. comparing between PRP, HA, CS, combination therapies, and a placebo has substantiated this finding, with PRP and PRP mixed with HA providing the most improvement in pain and function per WOMAC and VAS scores at 3, 6, and 12 months post-injection [24]. In two studies by Chang et al. and Filardo et al., PRP has shown a more appreciable effect on patients with minor to moderate knee OA, or K-L grade 1 to 3, compared to severe knee OA [51,52]. While there is robust clinical evidence that PRP injections provide symptomatic relief, especially in non-severe knee OA, proving the regenerative and positive disease-modifying effects of PRP is an area of ongoing investigation.

Wakayama et al. were able to provide some imaging evidence of cartilage regeneration by PRP injections, utilizing the whole-organ MRI score (WORMS) to assess cartilage integrity and synovial volume at medial femorotibial, lateral femorotibial, and patellofemoral joints. In their study of 161 patients who received PRP injections versus a historical control group of 30 patients who did not, 6 months post-PRP therapy led to a statistically significant improvement in the mean and total WORMS cartilage score for all three joints, with a reduction in synovial volume correlating with the improvement in KOOS [7]. However, their study is not prospective in design due to the different patient population used between the study and control groups. Furthermore, their sample size is limited, and much larger studies must be done to substantiate imaging evidence of cartilage regeneration via PRP therapy.

## 7. Bone Marrow Aspirate Concentrate

BMAC is different from PRP in that it is derived from bone marrow via harvesting from the posterior iliac crest, with the benefit of obtaining a formulation rich in MSCs and hematopoietic stem cells (HSCs) in addition to growth factors and other regenerative bioactive molecules. The bone marrow harvest is usually done under local anesthesia with a specialized needle utilized to aspirate bone marrow to about 60–90 mL. It is mixed with an anticoagulant to prevent clotting, is filtered, and undergoes density-gradient centrifugation to isolate a concentrate of MSCs, HSCs, and growth factors, which collectively form BMAC [53].

BMAC contains similar important bioactive molecules as PRP, such as TGF-β, PDGF, and VEGF. However, with the addition of MSCs within BMAC, those molecules promote chondrocyte differentiation of stem cells via TGF-β, proliferation of such cells and extracellular matrix deposition via PDGF, and nutrient delivery for lasting tissue repair via VEGF. Because of this, BMAC should have a higher potential to provide both symptomatic and structural changes in knee OA compared to PRP, which, thus far, has primarily provided symptomatic relief [54].

When looking primarily at symptomatic and functional improvement, BMAC has not been shown to be superior to PRP. In a prospective RCT by Anz et al., BMAC and PRP both resulted in an improvement of WOMAC and subjective International Knee Documentation Committee (IKDC) scores at 12 and 24 months in patients with K-L grade 1–3 knee OA. However, there was no notable difference between those two biologics at any time point [54]. El-Kadiry et al. were able to provide data that BMAC therapy does result in longer-lasting symptomatic improvement per VAS and WOMAC scores, even after 24 months, while PRP therapy resulted in a decline in improvement after 12 months. Other studies, however, could not reproduce such positive outcomes when BMAC was compared to PRP, HA, and/or the placebo [55]. It is important to note that there are inconsistent results across different RCTs, mainly due to an absence of standardized criteria for patient selection and uniform treatment protocols, limiting the reproducibility of outcomes.

As for structural changes, Kon et al. provided imaging evidence of a positive disease-modifying effect of BMAC when their study demonstrated both functional improvement and reduction in bone marrow edema on MRI with IA and subchondral BMAC injections [58]. Other studies have demonstrated a regenerative effect of BMAC on cartilage repair. In a study by Jin et al., patients undergoing high tibial osteotomy with microfracture (MFX) combined with BMAC for medial unicompartment knee OA have resulted in improved International Cartilage Repair Society (ICRS) scores compared to MFX alone (7.8 ± 3.1 vs. 6.0 ± 3.6, *p* = 0.035) [55,56]. In a study by Gobbi and Whyte, BMAC and HA combination therapy in full-thickness cartilage injury resulted in improved KOOS, IKDC, VAS, and Tegner Activity Scale (TAS) as well as enhanced cartilage quality visualized by repeat knee arthroscopies [55,57].

According to the most recent meta-analyses by Han et al. and Jawanda et al. in 2024, BMAC did provide symptomatic improvement per VAS, KOOS, and WOMAC for patients with knee OA, but it did not result in superiority over other injections, such as PRP [93,94]. Not enough studies utilized imaging modalities as assessment criteria to be considered an endpoint for the meta-analyses. While BMAC has some promising outcomes and potential, inconsistent clinical data across multiple RCTs continues to be an issue. Standardization of patient selection, preparation protocols, treatment dosage and plan, and assessment criteria is crucial in the reproducibility of outcomes [55]. Further evidence on a positive disease-modifying effect is required for BMAC to be considered an alternative to current treatment options for knee OA.

## 8. Stromal Vascular Fraction

SVF is a collection of cells from adipose tissue comprising adipose-derived stem cells (ADSCs), MSCs, endothelial precursor cells, leukocytes, smooth muscle cells, and pericytes [59]. They are obtained via liposuction from subcutaneous adipose tissue, commonly from the abdomen or thigh, followed by isolation of cells through enzymatic digestion. Collagenase type 1 is the most commonly used enzyme to digest the tissue matrix, resulting in a higher cell count. Alternatively, non-enzymatic or mechanical methods, such as vortexing, filtration, and fragmentation, can be used for faster processing but often result in lower yield of cells [60,95].

Researchers have recently focused on SVF as a potential alternative to BMAC due to the higher concentration of MSCs found in adipose tissue compared to bone marrow. In one review, they found that adipose tissue contains 500 times more MSCs than bone marrow for the same amount of tissue [60]. The hypothesis is that injecting a high concentration of MSCs and other stem cells in an inflammatory, high-mechanical-stress environment, such as the osteoarthritic joint, would result in appropriate differentiation of those cells, resulting in the regeneration of articular cartilage. In a small clinical trial of SVF therapy for six patients with grade I/II cartilage defects, Ren et al. found an improvement in the WOMAC pain and function subscale post-therapy at 12 and 24 weeks. They also utilized 3D MRI imaging of the knee pre- and post-SVF therapy for quantitative evaluation and found a significant increase in cartilage value in both the defect cartilage area and the whole cartilage area at 12 weeks. More specifically, the cross-sectional area and thickness of femoral and tibial cartilages were increased [8].

More recently, a prospective, single-arm cohort study by Boada-Pladellorens et al. with a larger sample size of 78 knee OA patients revealed similar results in terms of pain and functional improvement. Their study found improvement in VAS, all KOOS subscales, and SF-36, a quality-of-life survey, at 1, 6, and 12 months. However, their quantitative assessment via Magnetic Resonance Observation of Cartilage Repair Tissue (MOCART) values did not reach statistical significance in improvement pre- vs. post-SVF therapy [61]. Retrospective studies by Kim et al. and Mehling et al. also revealed improvement in pain and mobility up to 12 months with SVF therapy [62,63]. Kim’s study even found statistically significant improvement in MOCART values 12 months after SVF and arthroscopic treatments compared to arthroscopic treatment alone in patients with moderate to severe knee OA. It is possible that differences in MOCART changes between Boada-Pladellorens’s study and Kim’s study may be due to arthroscopic intervention and scaffold usage to apply SVF directly onto the cartilage defect sites. Unlike Boada-Pladellorens’s study, which used intra-articular injection of SVF under ultrasonography, Kim’s study utilized arthroscopic intervention followed by a fibrin glue product to be used as a scaffold for SVF implantation directly onto the cartilage defect site. Kim et al. hypothesized that because studies using cell tracking have found injected MSCs to be concentrated in other areas of OA joints, such as the synovium, as opposed to the cartilage defect site, it is important to implant SVF directly onto the defect lesion. This may explain the differences in MOCART results between those two studies [61,63].

A 2025 meta-analysis by Han et al. revealed that across 671 patients with nine RCTs, SVF resulted in an improvement in pain and function compared to placebo or HA at 3,6 and 12 months; however, it was inferior compared to CS at 3 months with no significant differences at 6 and 12 months. The authors note that a high heterogeneity in function scores is therefore not a reliable result [64].

With some clinical improvement and imaging evidence of cartilage regeneration, SVF therapy seems promising for patients with knee OA and cartilage defects. Given that SVF appears to be most efficacious when applied directly onto the cartilage defect site via arthroscopy, it does not eliminate the necessity of surgical intervention. There is a need for high-quality, reproducible RCTs to compare SVF to placement and to current standard treatment options. There must also be a standardized protocol across the studies in processing the SVF product, patient selection, application method, and assessment benchmarks.

## 9. Matrix-Based Orthobiologics

### Matrix-Induced Autologous Chondrocyte Implantation

MACI is a newer, more advanced version of ACI using synthetic biodegradable collagen membrane patches as a scaffold instead of autologous soft tissue, such as harvested periosteum. Because this patch is produced ex vivo, it can be trimmed to fit the cartilage defect, and the cultured chondrocytes can be seeded onto it prior to implantation. This allows the implantation process to be smoother and less invasive, done under arthroscopy as opposed to arthrotomy with harvested periosteum.

While MACI is more appealing on a technical aspect, a prospective randomized study by Bartlett et al. did not reveal its superiority over ACI with porcine-derived type I/III collagen as a cover on patients with symptomatic chondral defects. Both ACI and MACI treatments resulted in an improvement in the mean modified Cincinnati knee score (17.6 vs. 19.6, *p* = 0.32) as well as in the ICRS score in the arthroscopic assessment at the one-year follow-up (79.2% vs. 66.6%). The rate of graft hypertrophy was 6% for ACI vs. 9% for MACI, and the reoperation rate for both groups was 9% [65]. Colombini et al. have found that MACI was more utilized for mild to severe knee OA, or K-L grade 2 to 4, while ACI was used for K-L grade 1, with similar efficacy, resulting in stable clinical improvements and similar failure rate up to 11 years [47].

MACI, like ACI, has clinical evidence of providing improvement in pain and function in focal full-thickness chondral defects, leading to a delay in the need for total knee arthroplasty [47,65,66]. However, MACI is not supported as a treatment option for diffuse, generalized OA on both the technical and clinical levels. It is an option to consider in younger patients with chondral injury who experience symptoms refractory to conservative treatments.

## 10. Hyaluronic Acid Hydrogels

HA hydrogels are cross-linked networks of HA that form a mesh-like scaffold in the joint by trapping water. It is commonly created by using agents such as 1,4-butanediol diglycidyl ether (BDDE), divinyl sulfone (DVS), and carbodiimides to form covalent bonds between naturally occurring HA molecules [96]. Unlike MACI, which utilizes a true matrix with a load-bearing, cell-anchoring scaffold, HA hydrogel is primarily a viscoelastic scaffold with weaker mechanical strength than collagen [67]. It does, however, provide good bioactivity when anchored with cells and GF, such as MSCs or PRP, due to its mesh-like structure [68,69]. Similar to traditional HA injection, HA hydrogel injections provide mechanical support with joint lubrication and cushion on a weight-bearing joint, improving pain and function, especially in K-L grade 2–3 knee OA [70]. HA hydrogel has an additional benefit of remaining durable for an extended period of time due to its cross-linked nature [97]. It has also been shown to suppress chondrocyte senescence in knee OA by inhibiting the TLR-2/NF-κB signaling pathway, potentially delaying OA progression [98]. Furthermore, dihydrazide-modified or catechol-conjugated aldehyde-modified HA hydrogel is known to mimic the extracellular matrix environment, providing a natural environment for appropriate chondrocyte function and promoting chondrogenic differentiation of stem cells, as evidenced in animal models [69,71].

Due to its mimicry of an accommodating microenvironment coupled with excellent bioactivity, HA hydrogel can serve as an exceptional scaffold for many drugs, biomolecules, and cells. Common ones used are MSCs, cultured chondrocytes, BMPs, TGF-β, PDGF, and CS. When MSCs are anchored to HA hydrogel and injected into the joint, MSCs first differentiate into chondrocytes due to the biomechanical environment of OA, then HA specifically interacts with CD44 receptors on chondrocytes, resulting in further chondrogenesis [69,71]. It is important to note that CD44 is a common co-receptor for GFs and, therefore, is commonly expressed in cancer cells. Therefore, adequate cancer screening must be considered prior to utilizing HA-based biomaterials [99]. Other biomolecules anchored to HA hydrogel, such as PDGF and TGF-β, also promote chondrogenesis by recruiting local stem cells (PDGF) followed by chondrogenic differentiation of those cells (TGF-β) with HA acting as a medium for cells to adhere to [69].

Currently, there are a couple of clinical trials underway that surgically implant HA hydrogel as a scaffold for MSCs to treat knee OA. A study being done by Park et al., which is currently in phase I/II, revealed that a composite of culture-expanded allogenic human umbilical cord blood-derived mesenchymal stem cells (hUCB-MSCs) and HA hydrogels (CARTISTEM) being applied to an ICRS grade 4 cartilage defect site with K-L grade 3 knee OA resulted in improved pain and function with arthroscopic and MRI evidence of durable repair without osteogenesis and tumorigenesis at the 1-, 3-, and 7-year follow-up marks [73]. A multicenter RCT phase 3 trial by Lim et al. found similar results with surgical implantation of CARTISTEM, leading to improvement in ICRS grade at 48 weeks as well as pain and function improvement compared to the microfracture group at the 3- and 5-year follow-up [72]. It is important to note that these data are based on the surgical implantation of hUCB-MSC-HA hydrogel and that there is a lack of human clinical data on the injectable forms of this composite. However, HA hydrogel and its utilization as both viscosupplementation and a scaffold for biomolecule delivery is promising [69]. With more clinical data on both surgical implantation and injectables, HA hydrogel can be a competitive treatment option for knee OA.

## 11. Amniotic Membrane Matrix and Amniotic Suspension Allograft

An amniotic membrane matrix (AMM) is a collagen and GF-rich biological tissue derived from the placental inner lining that is commonly applied in wound care to promote healing and anti-inflammation. It is produced by collecting, processing, and sterilizing the inner layer of the placenta obtained from healthy screened donors via elective cesarean section delivery [74]. AMM has a variety of anabolic GFs, including TGF-α, TGF-β, bFGF, epidermal growth factor (EGF), PDGF, and anti-inflammatory proteins (IL-4, IL-10, and IL-1 receptor antagonist-1). AMM also contains HA and various tissue inhibitors of metalloproteinases (TIMP)-1,2,4. This makes AMM an appealing orthobiologic as these properties are naturally chondroprotective and anti-inflammatory, which can slow the degenerative process in OA [75].

In the case of knee OA, AMM is often suspended, micronized, and cryopreserved to be used as injectables, better known as amniotic suspension allograft (ASA) [76]. In a double-blind, randomized, prospective study done by Pill et al., they found that both ASA and CS injection provide initial pain and function improvement at 6 weeks for patients with K-L grade 3 and 4 OA. However, ASA provided more sustained pain relief and function at 1-year follow-up [75]. Another single-blind, randomized multicenter RCT revealed significant improvement in pain and function for patients with K-L grade 2 and 3 OA per VAS and KOOS through 12 months with ASA treatment compared to HA or a placebo. They did not find any statistical differences among ASA, HA, and the placebo in the X-ray measure in the index knee, immunoglobulins, C-reactive protein, or anti-HLA serum levels. Also, no differences in the number and types of adverse events were noted amongst the groups [77,78]. Gomoll et al. further found that patients who failed HA or saline placebo treatment had statistically significant improvement in pain and function per Outcome Measures in Arthritis Clinical Trials—Osteoarthritis Research Society International simplified responder criteria (OMERACT-OARSI) with ASA injections at 3, 6, and 12 months compared with the crossover baseline [79].

On a molecular level, it is not surprising that ASA treatment has higher efficacy potential compared to HA alone, given that ASA intrinsically contains HA along with various other anabolic and anti-inflammatory biomolecules that promote cartilage repair. However, there has not yet been any imaging, histological, or clinical evidence of regenerative or disease-modifying effects of ASA. Further investigation with objective assessment parameters, such as MRI, arthroscopic, or histological evaluation, must be used to substantiate the regenerative capabilities of ASA.

## 12. Micro-Fragmented Adipose Tissue

MFAT is similar to SVF in that it is a processed autologous adipose tissue via liposuction or mini-lipoaspiration, rich in MSCs, ADSCs, and GFs. MFAT is mechanically processed, as opposed to an enzymatic process for SVF; therefore, it retains the surrounding extracellular matrix. This provides natural cushioning and a scaffold while promoting cartilage repair in conditions like knee OA [80].

In a retrospective single-center cohort study by Maeda et al., SVF and MFAT treatments were compared for efficacy and duration in patients with K-L grade 1–4 knee OA. In their study, both groups exhibited notable improvements in knee extension angle, ROM strength, and KOOS without any significant differences between them. MRI T2 mapping also revealed significant cartilage quality improvement in both groups, with the MFAT group resulting in a superior improvement in specific lateral regions. The MFAT group also revealed greater improvement in knee flexion angle compared to the SVF group. As for duration of improvement, the SVF group responder rate declined over time, especially after 6 months, while the MFAT group revealed sustained improvement at 6 months and up to 12 months per OMERACT-OARSI. The authors hypothesize that the SVF group results in early, rapid symptom relief due to its robust anti-inflammatory effect, particularly attributable to M2 macrophages and cytokines, such as TGF-β and IL-10. As for prolonged relief observed in the MFAT group, the authors believe that the presence of intrinsic extracellular matrix allows for both structural preservation and sustained release of cytokines and GFs, promoting tissue repair [9].

Given that MFAT and HA both provide a cushion-like effect and promote preservation of the articulating joint, an RCT led by Molnar et al. compared those two injections in terms of symptom improvement and structural changes in knee OA. In their 1- and 6-month follow-up, they found that both the MFAT and HA group exhibited symptomatic and functional improvement, as measured by KOOS, WOMAC, and VAS. The MFAT group resulted in a more sustained and positive improvement across the 1- and 6-month follow-up mark as opposed to the HA group, which plateaued in the first month. The MFAT group specifically showed a statistically significant KOOS symptoms subscale at 6 months compared to HA, which can be attributable to the immunomodulatory effect of MFAT that is not present in HA. The authors also utilized delayed gadolinium-enhanced MRI of cartilage (dGEMRIC), which revealed greater glycosaminoglycan content in cartilage with the MFAT group, supporting its regenerative effect via MSCs, ADSCs, and GFs clinically [81].

When MFAT was compared to biologic fluids containing similar regenerative cells and GFs, such as PRP, no significant differences were found in terms of clinical outcomes. In a prospective RCT by Zaffagnini et al., both the MFAT and PRP groups resulted in clinical improvement as measured by IKDC and KOOS at the 6-month follow-up. No differences were found in terms of clinical outcomes, adverse events, and failures. Furthermore, there were no changes in X-ray or MRI findings pre- and post-injections for both groups. The authors did find that the MFAT group did reach minimal clinically important difference (MCID) per IKDC score at 6 months for more advanced knee OA compared to the PRP group [82]. Other studies that also compared MFAT and PRP with or without HA found similar non-superiority results between the two groups, even up to 24 months [100,101]. Given that MFAT has yet to prove its superiority over biologic fluids orthobiologics, it is currently considered another alternative therapy option.

## 13. Molecular-Based Orthobiologics

### Recombinant Fibroblast Growth Factor-18

rFGF-18 is an Escherichia coli-derived 20-kilodalton (kDa) protein, structurally analogous to human FGF-18, that plays a significant role in bone and cartilage homeostasis. More specifically, it induces proliferation of articular chondrocytes, resulting in increased synthesis of hyaline cartilage. In rat osteoarthritis models, rFGF-18, also known as sprifermin as a brand name, has been shown to increase knee joint cartilage thickness. Hochberg et al. started an FGF-18 osteoarthritis randomized trial with the administration of repeated doses (FORWARD), which is a 5-year, dose-finding multicenter RCT, currently in phase II of clinical trial. With a sample size of 549 patients with K-L grade 2 or 3 symptomatic knee OA between the age of 40–85 years old, they were divided into 5 groups with variable intra-articular sprifermin doses and regimen at 0 μg or placebo every 6 months (n = 108), 30 μg at every 6 months (n = 111) or 12 months (n = 110), and 100 μg every 6 months (n = 110) or 12 months (n = 110). Their primary endpoint was the difference in total femorotibial joint cartilage thickness as measured by quantitative MRI at 2 years. Secondary endpoints included pain and function changes at the 2-year mark, utilizing WOMAC scores. Amongst the 474 patients who completed a 2-year follow-up, the authors found a dose-dependent increase in the total femorotibial joint cartilage thickness, with 100 μg group exhibiting a 0.05 mm increase every 6 months (95% CI, 0.03–0.07 mm). However, they did not find any statistically significant differences in total WOMAC scores when compared across different groups. Therefore, the clinical importance of rFGF-18, when used alone, is currently uncertain despite regenerative change seen on imaging. As for adverse events, the most common treatment-emergent adverse events were arthralgia and back pain, which were observed across all 5 groups, including the placebo group, with similar incidence. No adverse event specifically attributable to sprifermin was noted [10]. There is a possibility that if rFGF-18 were to be injected with biocompatible agents that provide an anti-inflammatory effect, it can potentially provide both symptomatic relief as well as cartilage regeneration.

## 14. TPX-100

TPX-100 is a 23-amino acid peptide derived from matrix extracellular phosphoglycoprotein (MEPE) that has been shown to promote articular cartilage production in vitro and in vivo. MEPE is an important protein produced by osteoblasts and osteocytes for bone turnover, remodeling, and mineralization. It has been shown to be downregulated in OA cases. In goat models with full-thickness cartilage defect, TPX-100 resulted in an increased type II collagen and articular cartilage formation as early as 6 months, confirmed with immunostaining [12]. McGuire et al. completed a phase II double-blind, placebo-controlled RCT with 104 participants, 78 of whom were analyzed for quantitative femoral B-score and cartilage thickness via MRI 6 and 12 months after TPX-100 injection, as a primary efficacy outcome measure. Participant-reported outcomes, measured by WOMAC, KOOS, and numeric rating scale for pain (NRS), were documented as clinical outcome measures at 3, 6, and 12 months [11,12].

Participants had bilateral moderate to severe knee cartilage defects (ICRS grades 2–3), and each subject’s contralateral knee was given a placebo (saline) for the paired internal control. TPX-100 was first evaluated for safety with sequentially increasing doses at 25 mg, 50 mg, 100 mg, and 200 mg per injection for 6–9 subjects with no notable adverse effect. For TPX-100-treated knees (four weekly IA injections at 200 mg), they revealed a significant decrease in pathologic bone-shaped change compared to placebo-treated knees at 6 and 12 months. TPX-100-treated knees also exhibited a correlation between B-score changes and medial and total tibiofemoral cartilage thickness changes at 12 months, indicating that TPX-100 significantly delays pathological bone shape change and stabilizes cartilage up to 12 months. Furthermore, authors found statistically significant positive changes for both WOMAC and KOOS at 6 and 12 months with TPX-100-treated knees with decreased overall analgesic use, including NSAIDs [12]. Given both imaging evidence of delay in knee OA as well as positive clinical outcomes, TPX-100 is becoming a promising orthobiologic treatment for patients with cartilage defects. More studies with a longer follow-up duration are warranted to substantiate the currently limited evidence of this peptide.

## 15. Lorecivivint

LOR is a small-molecule Wnt pathway modulator that provides potential regenerative and positive disease-modifying effects in knee OA by inhibiting CDC-like kinase 2 and dual-specificity tyrosine phosphorylation-regulated kinase 1A (DYRK1A) within the Wnt pathway. In vitro studies have shown that such modulated Wnt signaling results in decreased catabolic proteases and increased extracellular matrix production by chondrocytes, reduced STAT3 and NF-κB signaling with decreased inflammatory cytokine production by synovium. In rat and dog models, no adverse effects were observed at approximately 400 times the intended dose in humans [42].

With promising potential and no recorded adverse effects, LOR recently underwent a 28-week phase 3 multicenter RCT by Yazici et al. with a single IA injection of 0.07 mg. This dose was previously determined to be most efficacious in phase 2 of the study when compared to other doses of 0.03 mg, 0.23 mg, and the placebo [42]. In their study, 498 patients with K-L grade 2–3 knee OA underwent a single IA LOR 0.07 mg injection with a follow-up at 12 weeks post-injection. When compared to the placebo, LOR failed to meet the primary endpoint of improvement in pain NRS. No significant differences were noted across other patient-reported outcomes, including WOMAC. Since 51.9% of the patients in this study had K-L grade 3 knee OA, the authors felt that LOR may be more effective in earlier stages of knee OA. They performed post hoc analysis specifically on patients with K-L grade 2 knee OA and found a statistically significant improvement in pain NRS at week 4. No adverse effects were noted [83].

The same authors then performed a 60-month observational extension study of their 12-month phase 2a trial and 6-month phase 2b trial, with a total of 584 patients diagnosed with K-L grade 2 or 3 knee OA. In their post hoc subgroup efficacy analyses, the LOR group revealed greater improvement in WOMAC pain and function compared to the placebo group up to 12 months out. No differences in joint space were noted on imaging. No treatment-related adverse effects were noted by the authors [102]. Despite seemingly mixed results between current phase 3 trial findings and observational extension study from phase 2a/2b, LOR remains a potential orthobiologics for treatment of early-stage knee OA, based on its mechanism of action and safety profile. More studies must be done to substantiate the efficacy of this medication with additional endpoints, including high-quality image assessment, such as MRI.

## 16. LNA043

LNA043 is a 26 kDa protein derived from angiopoietin-like 3 that is a known chondrogenic inducer for MSCs. LNA043 promotes chondrogenesis and cartilage production by binding to fibronectin receptor, integrinα_5_β_1_, on MSCs and chondrocytes. In vitro studies have confirmed that this results in chondrogenic differentiation of MSCs and increased cartilage matrix synthesis by chondrocytes. Following in vivo animal studies have confirmed such a mechanism of action with observable regeneration of hyaline articular cartilage in preclinical OA and cartilage defects. Given this regenerative potential, Gerwin et al. performed a phase I RCT with 28 patients with knee OA scheduled for TKR. These patients received one of five IA LNA 043 doses (0.2, 2, 10, 20, or 40 mg), with four patients per cohort, administered 7 days before the surgery. An additional 20 mg dose was given at 2 h or 21 days before the surgery. Only one patient reported mild transient dry mouth and dysgeusia thought to be related to the treatment. This was resolved before the completion of the study. No anti-LNA043 antibodies were detected in the patients’ serum [41].

During surgery, articular cartilage for each patient was biopsied and sent to the laboratory for immunohistochemical (IHC) staining. At 7 or 21 days before the surgery administration, LNA043 was not detectable by IHC in articular cartilage. However, administration 2 h before the surgery revealed detection of LNA043 in articular cartilage by IHC. LNA043 also penetrated four times deeper into the injured cartilage compared to the uninjured cartilage of the same joint. Furthermore, post hoc global transcriptomics profiling via RNA sequencing on injured and uninjured cartilage treated with LNA043 revealed downregulation of potential OA mediators, such as fibronectin (FN1), osteopontin (SPP1/OPN), delta/notch-like EGF repeat-containing transmembrane receptor (DNER), and osteoprotegerin (TNFRSF11B/OPG), and upregulation of cartilage matrix components, including matrilin-4 (MATN4) and collagen type IX (COL9A1). LNA043 also upregulated the expression of dickkopf-1 (DKK1) and frizzled-related protein (FRZB), which are known Wnt pathway inhibitor proteins [41].

Another study by Trattnig et al. revealed that treatment with four weekly IA LNA043 20 mg injections resulted in the regeneration of damaged femoral articular cartilage appreciable by MRI, when compared to a placebo, for up to 28 weeks. More specifically, a medial femoral region with cartilage damage had refilling detected over time, compared to no overgrowth in the lateral region. The safety profile overall was favorable, with some reported mild joint swelling and arthralgia compared to placebo [13].

Based on its in vitro and in vivo findings, LNA043 has promising potential in providing regenerative and positive disease-modifying effects on OA joints with minimal adverse effects. By affecting genetic expression, a single IA injection may provide sustained regenerative effects. As of writing this paper, this drug is in phase 2b trial [13,41].

## 17. Body Protection Compound-157

BPC-157 is a 15-amino acid peptide originally derived from human gastric fluid that has been shown to provide strong anti-inflammatory, angiogenic, collagen-producing, cell migrative, and proliferative properties that are useful in wound healing. Now synthesized chemically in a laboratory setting, BPC-157 provides these effects via extracellular signal-regulated kinase 1/2 (ERK 1/2), VEGF2-nitric oxide (NO), and focal adhesion kinase (FAK)-paxillin signaling pathways. The ERK 1/2 pathway promotes endothelial and muscle repair, induces angiogenesis and fibroblast activity, and exerts anti-inflammatory effects. VEGF2-NO pathway also promotes angiogenesis and fibroblast activity, while the FAK-paxillin pathway is responsible for fibroblast proliferation and collagen synthesis, making BPC-157 useful in musculoskeletal repairs and performance enhancement [84]. While BPC-157 is known to have a short half-life, often cited as less than 30 min, its effect in animal studies has been shown to persist for weeks to months [103,104,105,106]. These sustained effects are believed to be due to the activation of multiple gene expression pathways, as mentioned before, which can continue independently. Therefore, this peptide can potentially serve as a catalyst in triggering self-sustaining healing mechanisms present in our bodies without the need for recurrent injuries or inflammation [84].

In animal models, BPC-157 has been shown to be effective in tendon and ligament repair, muscle regeneration, bone healing, and performance enhancement [38]. However, human clinical trials are severely lacking. Especially when it pertains to utilizing BPC-157 as an IA knee injection, one study has been completed thus far. In a 2021 study, Lee and Padgett evaluated the effectiveness of BPC-157 and the combination therapy of BPC-157 and thymosin-beta-4 (TB4) in 16 patients with knee pain. Fourteen out of 16 patients reported significant pain relief with either BPC-157 or the combination therapy from 6 months to 1-year post-injection. However, this study has many limitations, including a lack of unifying diagnoses and control groups; therefore, it is very difficult to establish a cause-and-effect relationship and prove the mechanism of action behind the pain relief achieved by patients with potentially different diagnoses [38].

The lack of human clinical trials results in uncertainty for both the efficacy and safety profile of BPC-157. In three human clinical trials completed as of 2026, all with small sample sizes, none reported any notable adverse effects. However, the in vitro effects of BPC-157 are concerning for potential unregulated angiogenesis, which could result in the proliferation of tumor cells as well as the amplification of immune and inflammatory diseases [85,86]. BPC-157 is also concerning for the activation of the NO pathway, which can cause an inhibition of heme insertion into hemoglobin, resulting in altered activity of heme thiolate and cytochrome enzymes, affecting red blood cell formation and drug metabolism [87,88,89].

While BPC-157 has some potential as a regenerative molecular orthobiologic, more human clinical trials must be completed to prove the efficacy and safety of BPC-157 in musculoskeletal injuries and degeneration.

## 18. Discussion

Orthobiologics have garnered clinical and research interest in recent years due to utilizing the potential regenerative properties of stem cells and peptides to provide functional and symptomatic improvement in patients with knee OA. Depending on their composition and classifications, orthobiologics utilize pluripotent stem cells, GFs, cytokines, or peptides that alter genetic expressions to provide sustained regenerative effects. However, there are limitations that currently prevent them from being adopted as the mainstay treatment for musculoskeletal degenerative conditions like OA.

For cell-based, biologic fluids-based, and some matrix-based orthobiologics, variance in autologous sources in terms of age, health, and genetics can lead to inconsistencies in concentration and, therefore, efficacy of GFs, cytokines, and cells. This is compounded by the differences in protocols in acquiring, preparing, and administering these autologous orthobiologics across practitioners. This results in difficulty in achieving consistent and reproducible results clinically and in research across the patient population [5]. This limitation is not prevalent in molecular-based orthobiologics, as they are predominantly synthetic; therefore, they can be manufactured with high specificity and uniformity to be used clinically with consistent results.

A more notable limitation that applies to all types of orthobiologics currently is the lack of uniformity in inclusion criteria, treatment administration method, assessment criteria, and primary endpoints for long-term clinical studies. Particularly in knee OA studies, inclusion criteria for many orthobiologic studies varied across different K-L grades with varied numbers and sizes of cartilage defects, making it difficult to interpret results across studies. As for treatment administration methods, many studies are utilizing different concentrations of orthobiologics and control medications, as well as study-specific combination therapy without any replicated studies to verify their efficacy results. There are also inconsistent uses of assessment criteria and primary endpoints in both subjective patient-reported outcomes, such as WOMAC, KOOS, NRS, and VAS, and objective imaging or histological assessments, including X-ray, 3D MRI, dGEMRIC, WORMS, and cartilage biopsy with IHC, across studies. The lack of replicated studies with standardized criteria challenges the general clinical applicability of the data produced by current studies; therefore, it creates hesitancy in adopting orthobiologics as the mainstay treatment.

In addition to a lack of standardized clinical trials, there is also a limited number of large-scale RCTs, which makes it difficult to confidently assess the risks of the orthobiologics across different patient populations. In cases of ACI and MACI, there is always a risk of graft hypertrophy due to uncontrolled collagen synthesis by additional chondrocytes [46,47,48]. Variability in GF, cytokines, and stem cell yield across cell-based and biologic fluids-based orthobiologics may also result in inconsistent therapeutic results with unreliable inflammatory response [107]. Furthermore, the utilization of GF with stem cells has risks of unregulated cellular proliferation with angiogenesis leading to tumor formation [108]. There is a necessity in conducting large-scale multi-center RCTs across different patient populations to adequately identify the safety profile for each orthobiologic before adopting them clinically.

As clinicians and researchers deepen their understanding of molecular interactions between biologics and pathologic conditions, they continue to devise novel biologic therapies that would target that pathophysiology differently, with the hopes of more consistent efficacy, enhanced regenerative properties, and better safety profiles. With advancements in 3D bioprinting technology, some orthobiologic therapies, such as MACI, have replaced autologous tissues with synthetic materials, allowing therapies to be more universally applicable with less variability, yet still have the option for the materials to be individually customized for patients’ needs [36,109,110].

For other orthobiologics, for example, MSCs with GF mixture and HA hydrogel matrix, researchers even devised a combination therapy, CARTISTEM, as they hypothesize such agents may achieve a synergistic effect together in providing a sustained regenerative effect in osteoarthritis [72]. This type of creative, combinative approach results in vast possibilities in orthobiologic therapies, allowing multiple agents to target different aspects of pathophysiology at once, increasing the chance of long-lasting, reparative effects.

For certain molecular orthobiologics, such as LNA043, researchers were able to observe modification in specific genetic expression, which is beneficial in decelerating the progression of osteoarthritis [13,41]. This finding opens the possibility of utilizing gene-editing technology, such as clustered regularly interspaced short palindromic repeats (CRISPR), to provide specific genetic modifications, especially in stem cells, to augment regenerative and reparative effects for degenerative conditions [111].

With constant innovation for new agents, different combinatorial therapies, and possibilities of gene expression modification, the potential for orthobiologic therapy is very promising. However, because the potential, possibilities, and variabilities of orthobiologic therapies seem boundless, it is crucial to standardize and protocolize how these therapies are prepared and assessed clinically. Additionally, the preparation and administration of orthobiologics must be protocolized to eliminate variance across different practitioners. There must be a uniform assessment criterion that researchers agree upon and adopt as commonplace in evaluating clinical improvement with objective evidence of regeneration while accounting for potential adverse effects. This standardization will allow future clinical trials and studies of various orthobiologic therapies to be replicable, with the data becoming more applicable, comparable, and extrapolatable across the medical field. It will also allow meta-analyses of such studies to be more accurate. As singular orthobiologic therapies first become verified with increasing clinical evidence of efficacy and safety, then combinatorial therapies can be assessed with higher confidence.

## 19. Conclusions

Orthobiologics is a promising therapy for knee OA, unique in that it has the potential to provide a positive disease-modifying effect, which current treatment options do not offer. If it can deliver on its potential, orthobiologics can delay and even reverse the chronological progression of OA, allowing patients to have a better, more functional quality of life, even in their elderly years. It can also significantly reduce medical care burden and cost to the system by decreasing the need for frequent medications, imaging, and surgical care. While cell-based and biologic fluids-based orthobiologics have the most potential and clinical attention due to their utilization of autologous stem cells, they have more challenges in being adopted en masse due to the labor-intensive process in preparing the biologics. On the other hand, matrix-based and molecular-based have the advantage of being synthetic by nature, allowing them to be manufactured and distributed en masse with minimal labor. It also allows their future clinical studies to be easily standardized with consistent dosage and administration methods without the variability in concentration.

Once safety and efficacy are established and widely accepted, orthobiologics can be central in advancing the field of regenerative medicine. Its regenerative properties can potentially be applied to various musculoskeletal degenerations and injuries outside of osteoarthritis. Continuing to understand and expand on the mechanism of action of orthobiologics may also guide the engineering of novel regenerative therapies applicable beyond the field of orthopedics. The possibilities of translational findings that could arise from continued clinical research on orthobiologics are truly exciting for the future of regenerative medicine. It is inspiring many researchers and clinicians to push the boundaries of the field.

## Figures and Tables

**Table 1:** Classifications of orthobiologics and their examples.

Classes of Orthobiologics	Composition	Key Examples
Cell-based	Cells with regenerative properties	MSCs, ACI
Biologic fluids-based	Concentrations of regenerative cells and proteins	PRP, BMAC, SVF
Matrix-based	Allogenic, xenogeneic, or synthetic extracellular matrix	Matrix-induced autologous chondrocyte implantation (MACI), HA hydrogel, amniotic membrane matrix/amniotic suspension allograft (AMM/ASA), micro-fragmented adipose tissue (MFAT)
Molecular-based	Molecules, such as peptides, with regenerative properties and disease-modifying effects in OA	rFGF-18, TPX-100, LOR, LNA043, BPC-157

**Table 2:** List of key orthobiologics, their mechanism of action, and current clinical data.

Orthobiologics	Mechanism of Action	Current Clinical Data
MSC	IA supplementation of MSCs into the inflammatory OA joint increases chondrogenic differentiation with subsequent cartilage production [5]. Can be added with PTHrP and bFGF to control excessive chondrogenesis [43].	In vitro and ex vivo studies confirmed MOA. Multiple RCTs and meta-analyses confirmed symptomatic relief compared to placebo or HA, but there is no imaging or histological evidence of regeneration yet [5,44,45].
ACI	Surgically implanting autologous chondrocytes with a harvested patch of tissue, sewn over the cartilage defect, to augment cartilage regeneration over the site [46].	Currently level IV evidence with no RCTs. Mechanically only effective for a focal cartilage defect. No imaging or histological evidence of regeneration. Small risk of graft hypertrophy [46,47,48].
PRP	IA injection of autologous platelets and plasma rich in GF and cytokines to stimulate repair of damaged cartilage [49,50].	Multiple RCTs and meta-analyses confirmed symptomatic relief compared to placebo, HA, and CS, with some data on cartilage regeneration as evidenced by MRI [7,24,50,51,52].
BMAC	IA injection of autologous MSCs, HSCs, and GFs harvested from bone marrow to stimulate repair of damaged cartilage augmented by stem cells [53,54].	Multiple RCTs and meta-analyses confirmed symptomatic relief compared to placebo, but no reproducible studies that reliably prove superiority over HA, CS, or PRP [54,55,56,57]. Some data on cartilage regeneration as evidenced by MRI, but there is weak evidence currently [58].
SVF	IA injection of autologous ADSCs, MSCs, endothelial precursor cells, leukocytes, smooth muscle cells, and pericytes, enzymatically processed from adipose tissue to stimulate repair of damaged cartilage augmented by stem cells [8,59]. Similar idea to BMAC but higher yield of stem cells with lack of GFs [60].	Multiple studies revealed pain and functional improvement with SVF compared to placebo [8,61,62,63]. Although not consistent, some studies have shown cartilage regeneration via MRI, especially in cases of SVF applied directly to cartilage defect sites via scaffold usage and arthroscopy [8,62,63]. Meta-analysis confirms pain and functional improvement compared to placebo or HA, but there is no imaging evidence of regenerative properties [64].
MACI	ACI utilizes a synthetic collagen membrane instead of harvested autologous tissue. Less invasive than traditional ACI [65].	No strong evidence of superiority of MACI compared to ACI in efficacy [47,65,66]. Similar evidence level as ACI.
HA Hydrogel	IA injection of HA scaffold, providing both mechanical support of the joint and excellent bioactivity of anchored molecules, including stem cells, drugs, and GFs [67,68,69,70,71].	Several clinical trials, especially regarding CARTISTEM, have revealed the most potential for the treatment of knee OA. Currently in phase 3, CARTISTEM has demonstrated improved pain, function, and durable cartilage repair in patients with knee OA and cartilage injury [72,73]. No strong evidence of the injectable form of HA hydrogel in the treatment of knee OA.
AMM/ASA	Surgical implantation (AMM) or IA injection (ASA) of collagen and GF rich amniotic product to promote anabolic and anti-inflammatory effects on degenerative joints [74,75,76].	Few RCTs support prolonged pain and functional improvement up to 12 months with ASA compared to CS, HA, or placebo. No differences in X-ray measurements or inflammatory markers in serum [75,77,78,79].
MFAT	Similar to SVF but mechanically processed adipose tissues instead of the enzymatic process for SVF. Rich in MSCs, ADSCs, and GFs [80].	One RCT revealed prolonged pain and functional improvement with MFAT compared to SVF, with superior improvement in cartilage quality per MRI [9]. Another RCT demonstrated more sustained pain and functional improvement of MFAT compared to HA [81]. No differences when compared to PRP [82].
rFGF-18	IA injection of peptide analogous to FGF-18, which induces chondrocyte proliferation with increased hyaline cartilage synthesis [10].	Phase 2 clinical trial revealed an increase in femorotibial joint cartilage thickness in a dose-dependent manner of rFGF-18 measured by MRI at the 2-year mark. No notable pain and functional improvement noted. No treatment-specific adverse effect noted [10].
TPX-100	IA injection of a peptide derived from MEPE that induces articular cartilage production [11,12].	Phase 2 clinical trial demonstrated stabilized tibiofemoral cartilage thickness and a decrease in pathologic bone-shaped changes in TPX-100-treated knee compared to the contralateral OA knee, with a good safety profile. Functional and pain improvement was also noted up to 12 months compared to placebo [11,12].
LOR	IA injection of a small molecular Wnt pathway modulator that decreases catabolic proteases and inflammatory cytokine production and increases extracellular matrix production [42].	Phase 3 clinical trial with mixed results in pain and functional improvement when compared with placebo. Based on post hoc analysis, the authors note LOR might be more efficacious in earlier stages of knee OA. No imaging evidence of regenerative capabilities [42,83].
LNA043	IA injection of a peptide that induces chondrogenesis and cartilage production via fibronectin receptor. It has been shown to induce genetic expression favorable in delaying OA changes [41].	Phase 1 clinical trial revealed a mild transient case of dry mouth and dysgeusia, but otherwise considered safe. No immunogenicity noted. Positive cartilage penetration per IHC staining and post hoc global transcriptomics profiling demonstrated OA process-altering gene expression [41]. Weak imaging evidence of cartilage regeneration [13]. Unknown clinical importance based on current data.
BPC-157	IA injection of peptide that affects ERK 1/2, VEGF2-NO, and FAK-paxillin signaling pathways, resulting in increased angiogenesis, fibroblast activity, collagen synthesis, and anti-inflammation [84].	Very limited human studies in application to knee OA treatment. One study revealed improvement in knee pain with the BPC-157 combination, with TB4, but without a known diagnosis, or compared with the control group. Animal model studies have shown evidence of musculoskeletal repair [38]. Concern for safety profile, especially in altered drug metabolism, red blood cell formation, tumor cell proliferation, and exacerbation of immune and inflammatory diseases [85,86,87,88,89].

## Data Availability

No new data were created or analyzed in this study. Data sharing is not applicable to this article.

## Abbreviations

ACI	autologous chondrocyte implantation
ADSC(s)	adipose-derived stem cell(s)
AMM	amniotic membrane matrix
ANGPTL-3	angiopoietin-like 3
ASA	amniotic suspension allograft
BMAC	bone marrow aspirate concentrate
BMP-2	bone morphogenetic protein-2
BPC-157	body protection compound-157
CARTISTEM	composite of allogeneic hUCB-MSCs + HA hydrogel (product name used in cited trials)
CLK2	CDC-like kinase 2
COX	cyclooxygenase
CRP	C-reactive protein
CRSPR	clustered regularly interspaced short palindromic repeats
CS	corticosteroid
CV	cardiovascular
dGEMRIC	delayed gadolinium-enhanced MRI of cartilage
DKK1	dickkopf-1
DNER	delta/notch-like EGF repeat-containing transmembrane receptor
DYRK1A	dual-specificity tyrosine phosphorylation-regulated kinase 1A
EGF	epidermal growth factor
ERK 1/2	extracellular signal-regulated kinase 1/2
FAK	focal adhesion kinase
FGF/bFGF	fibroblast growth factor/basic fibroblast growth factor
FN1	fibronectin
FRZB	frizzled-related protein (FRZB)
GF(s)	growth factor(s)
GI	gastrointestinal
HA	hyaluronic acid
hUCB-MSCs	human umbilical cord blood–derived mesenchymal stem cells
HSC(s)	hematopoietic stem cell(s)
IA	intra-articular
ICRS	International Cartilage Repair Society
IHC	immunohistochemistry/immunohistochemical
IGF	insulin-like growth factor
IKDC	International Knee Documentation Committee
IL	interleukin
JSN	joint space narrowing
K-L	Kellgren–Lawrence (radiographic grading system for OA severity)
KOOS	knee injury and osteoarthritis outcome score
Knee OA	knee osteoarthritis
LNA043	angiopoietin-like 3–derivative LNA043 (cartilage-regeneration candidate)
LOR	lorecivivint
MACI	matrix-induced autologous chondrocyte implantation
MATN4	matrilin-4
MCID	minimal clinically important difference
MEPE	matrix extracellular phosphoglycoprotein
MFAT	micro-fragmented adipose tissue
MFX	microfracture
MMP(s)	matrix metalloproteinase(s)
MOA	mechanism of action
MRI	magnetic resonance imaging
MSC(s)	mesenchymal stromal cell(s)
NF-κB	nuclear factor kappa B
NO	nitric oxide
NRS	numeric rating scale
NSAIDs	nonsteroidal anti-inflammatory drugs
OA	osteoarthritis
OMERACT-OARSI	Outcome Measures in Rheumatology–Osteoarthritis Research Society International
OPG	osteoprotegerin
OPN	osteopontin
PDGF	platelet-derived growth factor
PGE2	prostaglandin E2
PRP	platelet-rich plasma
PTHrP	parathyroid hormone–related peptide
RANKL	receptor activator of nuclear factor κB ligand
RCT	randomized controlled trial
RFA	radiofrequency ablation
rFGF-18/rrFGF-18	(recombinant) fibroblast growth factor-18
ROM	range of motion
SF-36	36-item short form health survey
SPP1	secreted phosphoprotein 1
STAT3	signal transducer and activator of transcription 3
SVF	stromal vascular fraction
TAS	Tegner activity scale
TB4	thymosin beta-4
TGF-β	transforming growth factor beta
TIMP	tissue inhibitor of metalloproteinases
TKR	total knee replacement
TLR-2	toll-like receptor 2
TNF-α	tumor necrosis factor alpha
TNFRSF11B	tumor necrosis factor receptor superfamily member 11b
TRPV1	transient receptor potential vanilloid 1
TSG6	TNF-α–induced protein 6
VAS	visual analog scale
VEGF	vascular endothelial growth factor
Wnt	wingless/int-related signaling pathway
WOMAC	Western Ontario and McMaster universities osteoarthritis index
WORMS	whole-organ magnetic resonance imaging score
